# Acoustics and aerodynamic effects following glottal and infraglottal medialization in an excised larynx model

**DOI:** 10.1007/s00405-024-08519-x

**Published:** 2024-02-29

**Authors:** Liran Oren, Alexandra Maddox, Charles Farbos de Luzan, Changchun Xie, Rebecca Howell, Gregory Dion, Ephraim Gutmark, Sid Khosla

**Affiliations:** 1 Department of Otolaryngology-Head and Neck Surgery, University of Cincinnati, Cincinnati, OH USA; 2 Department of Aerospace Engineering and Engineering Mechanics, University of Cincinnati, Cincinnati, OH USA; 3 Department of Environmental and Public Health Sciences, University of Cincinnati, Cincinnati, OH USA

**Keywords:** Unilateral vocal fold paralysis, Medialization laryngoplasty, Implant location, Vocal efficiency, Intraglottal flow

## Abstract

**Objective:**

This study aimed to investigate the impact of the implant’s vertical location during Type 1 Thyroplasty (T1T) on acoustics and glottal aerodynamics using excised canine larynx model, providing insights into the optimal technique for treating unilateral vocal fold paralysis (UVFP).

**Methods:**

Measurements were conducted in six excised canine larynges using Silastic implants. Two implant locations, glottal and infraglottal, were tested for each larynx at low and high subglottal pressure levels. Acoustic and intraglottal flow velocity field measurements were taken to assess vocal efficiency (VE), cepstral peak prominence (CPP), and the development of intraglottal vortices.

**Results:**

The results indicated that the implant's vertical location significantly influenced vocal efficiency (*p* = 0.045), with the infraglottal implant generally yielding higher VE values. The effect on CPP was not statistically significant (*p* = 0.234). Intraglottal velocity field measurements demonstrated larger glottal divergence angles and stronger vortices with the infraglottal implant.

**Conclusion:**

The findings suggest that medializing the paralyzed fold at the infraglottal level rather than the glottal level can lead to improved vocal efficiency. The observed larger divergence angles and stronger intraglottal vortices with infraglottal medialization may enhance voice outcomes in UVFP patients. These findings have important implications for optimizing T1T procedures and improving voice quality in individuals with UVFP. Further research is warranted to validate these results in clinical settings.

## Introduction

Unilateral vocal fold paralysis (UVFP) frequently results in a lateralized, bowed vocal fold on the affected side. During phonation, the lack of adductor muscle function and tone results in incomplete glottic closure leading to a soft, breathy voice that can be difficult to understand in noisy environments [1]. Common UVFP treatments aim to permanently medialize the paralyzed vocal fold, so the adduction of the non-paralyzed fold will result in the prephonatory glottal shape (i.e., closed glottis). The paralyzed fold can be pushed medially by injecting a dermal filler, generally as a trial or temporary solution, or a Type 1 Thyroplasty (T1T) procedure employing a silastic block or Gore-Tex folded strip [2–4]. These surgical approaches improve laryngeal function even if complete glottal closure is not achieved because they reduce the glottal gap [5]. T1T procedures preserve a normal mucosal wave pattern during phonation [5]. The mucosal wave is an essential characteristic of the fold vibration and subsequent phonation that can be broken into two main modes: the first travels in the medial–lateral direction, and the second travels in the inferior-superior direction [6–8].

The vertical mucosal wave plays an essential role in creating the convergent-divergent glottal shape during the opening and closing phases of the fold vibrations [9]. The glottal shape determines the aerodynamic pressure distribution (i.e., force) acting on the walls. In the opening phase, the glottis resembles a converging nozzle, and the glottal airflow is attached to the entire medial surface of the vocal folds. During this phase, the intraglottal pressure distribution follows Bernoulli’s principle—it decreases as the size of the glottal opening decreases in the vertical direction. In the closing phase, the glottis takes on a divergence shape. The glottal flow, however, cannot follow the entire medial surface if the diverging angle exceeds a certain threshold value. When this occurs, flow is entrained into the void that is formed between the glottal jet and the medial wall. This entrainment flow can roll into a vortex that does not convect downstream and remains inside the glottis. These intraglottal vortices are thought to augment the negative pressure (i.e., pressure less than atmospheric) near the superior aspect of the fold, which can act as an additional (pulling) force during the closing phase [10–12].

While the glottal shape is created by a phase delay in the inferior-superior direction of the mucosal wave, the magnitude of the converging/diverging angle depends on the subglottal pressure (Psg) and the tissue biomechanical properties. The Psg value, which signifies the lung pressure or the vocal effort, imposes the extent of the lateral force that acts on the medial wall to displace the fold. The reaction to that force varies along the glottal height due to the inferior-superior tissue stiffness gradient [13–15]. In previous studies using an indentation test to characterize this gradient showed that the superior edge was about as stiff as the inferior edge at small displacement values (i.e., low strains). However, the inferior edge became much stiffer than the superior edge as the displacement increased due to the conus elasticus location [13, 16, 17]. Hence, the superior aspect of the medial wall can displace further than its inferior for a given force, which translates to a greater maximum divergence angle (i.e., greater vertical mucosal wave).

Although previous studies have focused on the implant shape [18–20], locations [19–22], material [23–27], and whether to include arytenoid adduction [26–29], no optimal technique has been identified and revision rates for T1T are as high as 12–25% [30, 31]. Furthermore, the effect of the implant's vertical location after T1T remains undetermined. The motivation for the current study is based on anecdotal reports by laryngologists of better voice outcomes when placing the implant more inferiorly. We hypothesize that medializing the paralyzed fold at the infraglottal level maintains the naturally occurring inferior-superior stiffness gradient resulting in greater divergence than a glottal-level medialization.

## Methodology

In short, measurement data were taken in six excised canine larynges. All cartilage and soft tissue above the vocal folds were removed, and the trachea was kept about 5 cm long. The larynx was positioned using three-prong support inserted into the arytenoid’s lateral surface and used for medial–lateral translation of the vocal processes. Phonation was induced by controlling the conditioned airflow supplied to the larynx. Acoustic measurements were taken using a 0.5-inch free-field microphone (model 4950, Bruel &Kjaer) placed approximately 30 cm laterally and superiorly to the glottal exit.

Preoperative data were collected in each larynx for a baseline and a UVFP case. The former was done by adducting both folds to the midline using the prongs inserted into the vocal processes. The latter was achieved by adducting one fold while the other was left in its natural rest position (simulating a paralyzed fold). In all cases (preoperative and operative), measurements were taken at low and high subglottal pressure (Psg). Low Psg was defined as 2–5 cmH2O above the phonation threshold pressure (PTP). High Psg was defined as 5–10 cmH2O above the low Psg value.

**Fig. 1:**
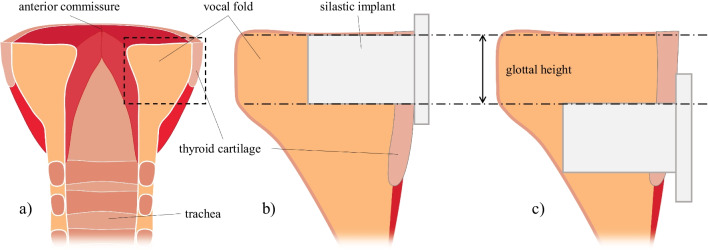
Schematic showing the implants’ approximate location. **a** Coronal plane. **b** Glottal. **c** Infraglottal

A T1T was performed by a laryngologist for the operative cases using Silastic implants. In each larynx, the implant location was tested in two places (Fig. 1). The first insert location was in the glottal level (hereafter, glottal implant), causing a uniform medialization of the inferior and superior edges of the paralyzed fold. After taking the measurements, the implant was removed and placed in a second location on the same paralyzed fold, approximately 2 to 3 mm lower (hereafter, infraglottal implant). The measurements were repeated using similar Psg values from the glottal case of that larynx. Hence, each larynx was tested eight times: 2 preoperative cases (baseline/UVFP) × 2 implant locations (glottal/infraglottal) × 2 Psg (low/high).

In one larynx (L4), the acoustic data were collected over its full phonation range in one continuous sweep. The sweep procedure gradually increased Psg (at 2 cmH2O increments from its PTP level) until phonation became dysphonic. It is worth noting that the dysphonic phonation occurred at different levels of Psg; therefore, the size of the data array varied for each case.

In three of the excised larynges (L4–L6), intraglottal flow velocity field measurements were also taken using particle image velocimetry (PIV). This technique can give spatial and temporal information on the glottal jet measured in the mid-membranous plane. The data from PIV measurement was used to calculate the circulation strength of the intraglottal vortex that developed during the closing phase of phonation. Circulation quantifies the amount of rotation inside an enclosed region and is defined as: $\Gamma = \int_{s} \omega_{z} ds$, where ${\omega }_{z}$ is the vorticity normal to the PIV plane, and $s$ is the surface area encompassing the vortex. The (maximum) magnitude of the glottal divergent angle during the closing phase was also extracted from the PIV data.

Other outcome measures used to compare between cases included vocal efficiency (VE) and the cepstrum peak prominence (CPP). The VE was calculated as the ratio of the radiated acoustic power to the aerodynamic power [32]. In this classic definition, $VE=\frac{2\pi {R}^{2}{10}^{-12}{10}^{\varvec{SPL}/10}}{{\varvec{P}}{\varvec{s}}{\varvec{g}}*{\varvec{Q}}},$ where R is the distance from the microphone to the sound source, SPL is the sound pressure level, Q is the mean glottal flow rate, and Psg is the subglottal pressure. CPP was calculated using the methodology outlined in the literature [33]. A fixed-effect model was used to assess the statistical association of VE and CPP (dependent variables) to the fixed effects of Psg and the experimental configurations.

## Results

**Fig. 2:**
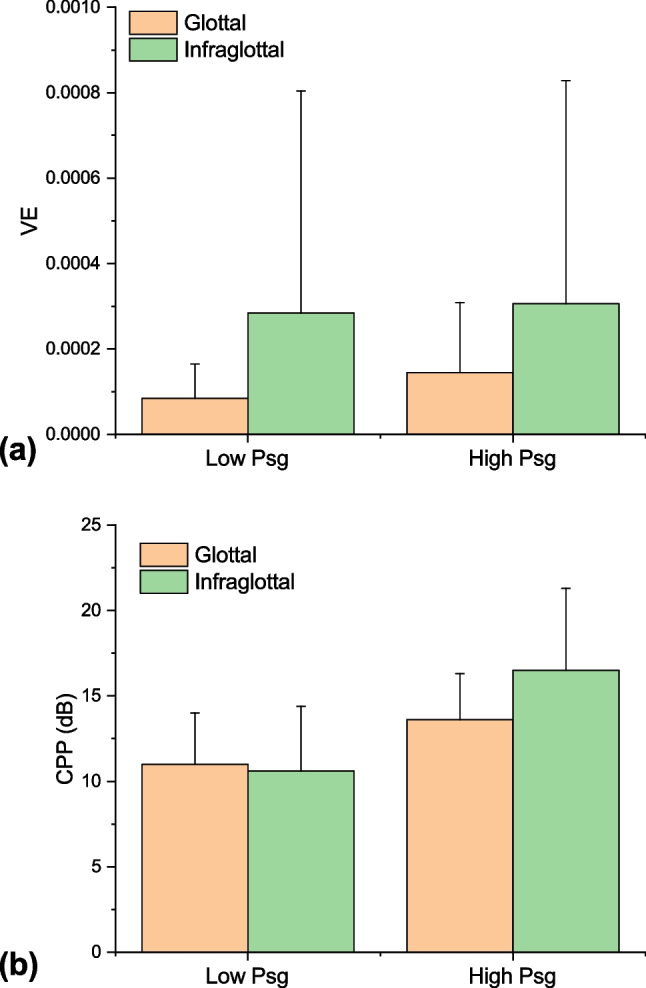
Change in **a** vocal efficiency (VE) and **b** cepstral peak prominence (CPP) based on implant location. The low- and high-subglottal pressure data from each larynx are averaged together

The VE and CPP data from each larynx are detailed in Table 1 and are summarized as mean plots for low- and high Psg (Fig. 2). These data show that the difference in implant location is statistically significant (*p* = 0.045) for VE. Except in one larynx (L5), VE was consistently higher with the infraglottal implant. The effect on CPP was not as pronounced. In each larynx, the CPP values were typically higher with the infraglottal implant, except at low Psg in L2 and L5, and high Psg in L6. Overall, the difference in CPP values between the glottal and infraglottal implants was not statistically significant (*p* = 0.234).

**Table 1:** Summary of outcome measures for each larynx implant location

Larynx	Glottal					Infraglottal				
	Psg (cmH_2_O)	VE	CPP (dB)	Max divergent angle	Γ (m^2^/s)	Psg (cmH_2_O)	VE	CPP (dB)	Max divergent angle	Γ (m^2^/s)
**1**	16.6	4.34E − 05	11.7			16.3	5.08E − 05	13.7		
	19.9	8.09E − 05	12.5			22.3	8.40E − 05	13.7		
**2**	11.3	7.20E − 05	12.5			11.7	1.39E − 04	10.2		
	16.0	9.49E − 05	18.0			16.6	1.88E − 04	18.7		
**3**	20.7	1.24E − 04	6.8			19.4	1.34E − 03	9.8		
	26.4	4.64E − 04	11.2			25.3	1.37E − 03	13.6		
**4**	12.7	9.05E − 06	14.4	14	7.22E + 02	15.8	3.76E − 05	16.2	50	5.48E + 03
	16.3	1.16E − 05	15.5	23	2.26E + 03	19.8	7.94E − 05	23.9	63	3.03E + 04
**5**	13.0	2.28E − 04	12.5	11	0.00E + 00	13.0	8.00E − 05	5.9	20	3.17E + 02
	16.4	1.61E − 04	13.5	12	3.95E + 00	18.7	6.06E − 05	18.6	23	3.95E + 03
**6**	13.4	3.08E − 05	7.9	11	7.83E + 00	13.0	3.50E − 05	8.0	21	2.31E + 03
	18.0	5.75E − 05	10.8	19	5.40E + 01	17.4	4.10E − 05	10.6	32	1.80E + 04

*Psg* subglottal pressure, *VE* vocal efficiency, *CPP* cepstral peak prominence, Γ – circulation

**Fig. 3:**
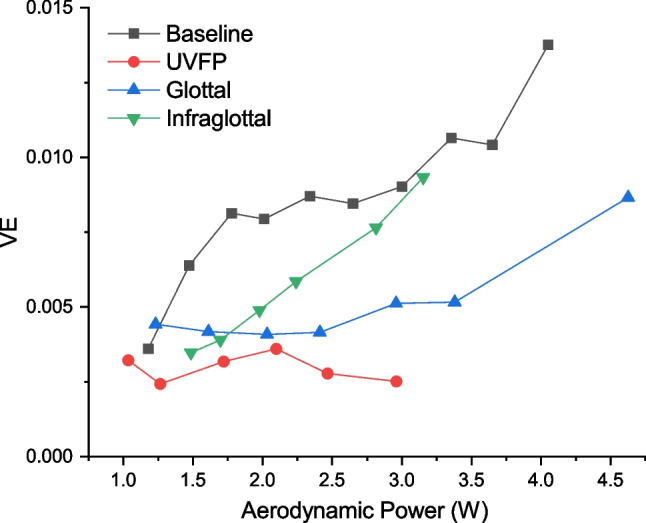
The vocal efficiency of a glottal implant, infraglottal implant, baseline, and phonation with UVFP over their full range of phonation. Aerodynamic power = Psg x Qmean

Plotting VE over the full range of Psg in one larynx (L4, Fig. 3) further illustrates the difference between implant locations. At low levels of aerodynamic power (defined as Psg multiplied by Q), VE is slightly higher with an infraglottal implant than with a glottal implant. As the aerodynamic power increases, so does the difference in VE between infraglottal and glottal implants. Furthermore, an infraglottal implant has a similar steep increase in VE (i.e., slope) as the baseline phonation. In contrast, a glottal implant shows a much smaller change when the aerodynamic power is increased.

**Fig. 4:**
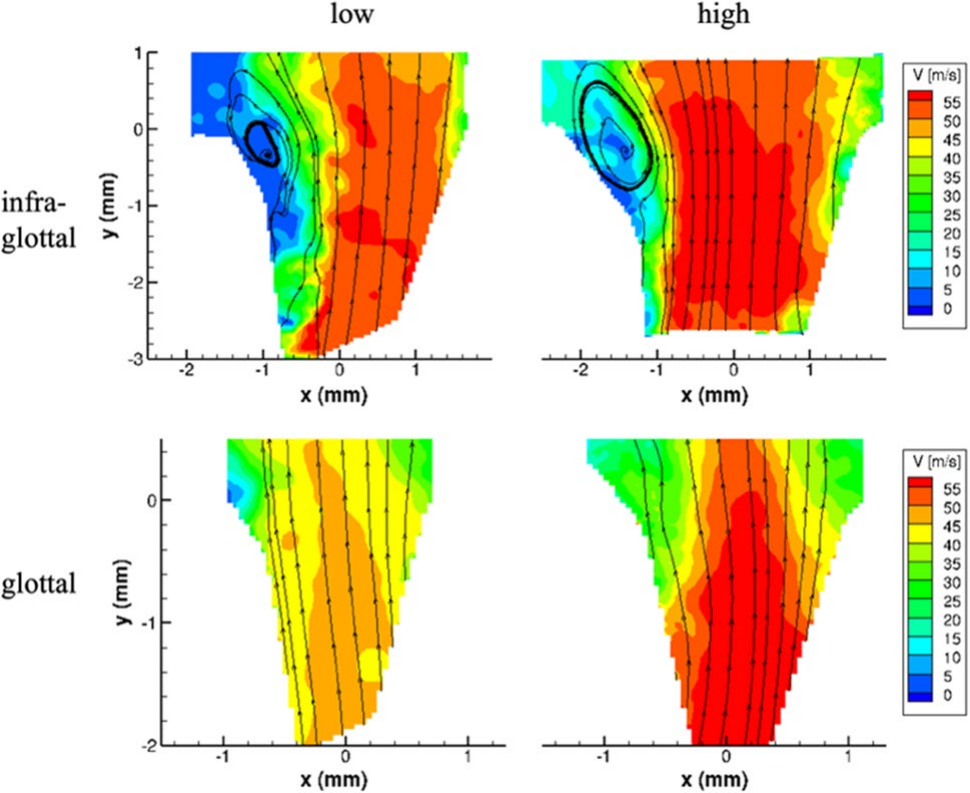
Intraglottal PIV velocity fields from one larynx (L4). Implants were placed in the left fold for all four cases. Infraglottal implant velocity fields (top row) show phase with maximum circulation strength. Glottal implant flow fields show phase with maximum divergence angle since circulation strength was always zero

The intraglottal velocity fields are shown in one larynx (L4) for the closing phases when the highest glottal circulation is computed (Fig. 4). The rainbow colors indicate contour levels based on the velocity magnitude. In general, warm colors mark the glottal jet, and cold colors imply entrainment flow. These contours show a larger glottal divergence angle with the infraglottal implant, enabling more entrainment flow to feed the vortex that develops between the wall and the glottal jet. This observation is shown qualitatively by plotting the flow's streamlines and observing that larger vortices are formed with the infraglottal implant. Larger divergence angles with infraglottal medialization were observed in other larynges, and these vortices' circulation strength also increased correspondently (Table 1).

## Discussion

Results from the current study show that the implant’s vertical location significantly affects VE, while the CPP measure is largely unaffected. In patients, lower CPP correlates with an increased perception of breathiness [33, 34]. The measure of CPP is correlated with glottal gap [35–37], which is accentuated in UVFP. This glottal gap, however, was closed in both implant locations, presumably not affecting CPP. Clinically, this observation suggests that the implant’s vertical location will not affect the perception of breathiness.

The change in VE has clinical implications in that many UVFP patients report symptoms of decreased loudness, increased vocal effort, and decreased intelligibility in noise [38]. VE is defined as the ratio of acoustic power over aerodynamic power, and increased VE means a louder voice for the same effort (or the same vocal intensity but with a lower effort). The current results suggest that larger divergent angles during closing would facilitate higher VE. Similarly, intelligibility in noisy environments has been shown to correlate with acoustic energy in higher harmonics [39]. Zhang and Chhetri [40] used a 2 mm wooden stick to medialize at different vertical locations. They observed increased acoustic energy in higher harmonics with inferior medialization, consistent with the current findings of an increased VE. Together, these findings support the anecdotal observation that infraglottal medialization yields better voice outcomes.

The slight difference in glottal shape is the likely mechanism to improve voice outcome. Zhang and Chhetri [40] suggested that inferior medialization increases the vertical height of the vibrating glottis, causing the acoustic energy to increase in the higher harmonics. This argument, however, seems unlikely because there is no evidence that the infraglottis tissue vibrates. Furthermore, the height of the medial glottal wall can be assessed from the PIV images, and it seems to remain the same regardless of the implant location for the three larynges we tested. On the other hand, an infraglottal implant enables a larger divergence angle to form during closing compared to a glottal implant at similar Psg.

A large divergence angle during closing is an essential feature because it will increase the strength of the intraglottal vortices. These vortices produce negative pressure near the superior aspect of the glottis, acting as an additional suction force, causing the folds to close faster. This increase in closing speed causes an increase in the maximum flow declination rate (MFDR), which is known to be correlated with increased SPL and energy in the higher harmonics. Previous computational models [12, 41] and experimental studies in excised canine larynges [11, 14, 42] have shown a strong correlation between the strength of these vortices, the divergence angle, MFDR, and VE.

The vertical stiffness gradient, which sets the divergence angle’s magnitude, is not constant but rather a function of the fold’s lateral displacement. The latter depends on the Psg magnitude. At low Psg, the difference in VE between implants is small as the stiffness gradient is similar in both cases yielding similar glottal divergence angle magnitudes. Placing a glottal implant likely stiffens both the inferior and superior edges, thus, minimizing the stiffness gradient and, consequently, the divergence angle. The infraglottal implant, however, predominantly stiffens the inferior edge, maintaining the stiffness gradient and resulting in larger divergence angles. Thus, at higher Psg, the difference in divergence angles is more pronounced. Clinically it suggests the change in VE is likely not significant for soft voices but is important for louder voices. This point is important because some patients with T1T still report difficulty projecting their voices.

The current study’s main limitation is not accounting for residual innervation or atrophy of the paralyzed fold. We also cannot accurately model the innervation of the non-paralyzed side. In addition, no vocal tract was used in this study.

A final caveat about the clinical implications of the current findings is that there can be a relatively large space between the vocal fold and inner perichondrium in atrophic folds. This dead space needs to be replaced by the implant. We recommend that the superior aspect of the fold should not be medialized once the dead space is eliminated, as the additional forces on the tissue from the implant may result in implant extrusion.

## Conclusion

In conclusion, this study explored the impact of the implant’s vertical location during T1T on acoustics and glottal aerodynamics. The results demonstrated that the vertical location of the implant significantly influenced VE, while the CPP remained less affected. The findings suggest that placing the implant inferiorly in the infraglottal region yielded better voice outcomes, which aligns with anecdotal reports by laryngologists. The study also highlighted the role of glottal shape and divergence angle in improving voice outcomes. Infraglottal implants enabled larger divergence angles during the closing phase, enhancing intraglottal vortices and subsequently affecting glottal closure. These findings provide valuable insights for clinicians performing the T1T procedure and underscore the importance of considering the implant’s vertical location for optimizing voice outcomes in patients with UVFP.

## Data Availability

Not applicable.
